# A case report of intracardiac bone cement embolization after posterior decompression and cement-enhanced pedicle screw fixation for osteoporosis and lumbar degeneration

**DOI:** 10.1097/MD.0000000000028826

**Published:** 2022-02-25

**Authors:** Kui Sun, Fuli Huang, Biru Liang

**Affiliations:** a Department of Orthopedics, Zhongshan Hospital of Traditional Chinese Medicine, Zhongshan, Guangdong, China; b Department of Spine Surgery, the Fifth Affiliated Hospital of Southern Medical University, Guangzhou, Guangdong, China.

**Keywords:** bone cement leakage, bone cement-enhanced pedicle screw fixation, intracardiac embolism, osteoporosis

## Abstract

**Rationale:**

Bone cement leakage is a common complication of percutaneous kyphoplasty (PKP) and percutaneous vertebroplasty (PVP) surgery and has also been reported in posterior decompression and cement-enhanced pedicle screw fixation. When bone cement leaks through the venous system, it will have serious consequences and even endanger the life of the patient, especially when the bone cement causes intracardiac embolism.

**Patient concerns:**

A 70-year-old woman developed chest tightness and decreased blood oxygen saturation following posterior decompression and cement-enhanced pedicle screw fixation.

**Diagnosis:**

After the patient was given symptomatic treatment, the symptoms were not relieved, the high-sensitivity troponin I level continued to rise, the electrocardiogram results were abnormal, and chest computed tomography (CT) revealed multiple flaky and strip-shaped dense shadows in the heart.

**Intervention:**

The patient underwent removal of foreign bodies from the heart under cardiopulmonary bypass and tricuspid valvuloplasty, removal of intracardiac bone cement, and repair of the tricuspid valve and chordae.

**Outcome:**

The patient recovered well postoperatively and was discharged from the hospital after 3 weeks. There were no intracardiac foreign bodies observed on chest CT after the operation.

**Lessons:**

For patients with cardiopulmonary discomfort after posterior decompression and bone cement-enhanced pedicle screw fixation, in view of the limitations of radiographic examination, we recommend performing chest CT examination to confirm the diagnosis. For patients with intravascular foreign body embolism, multidisciplinary team joint treatment saves lives.

## 1 Introduction

Posterior interbody fusion and internal fixation, as common surgical procedures for the treatment of osteoporosis and lumbar degenerative diseases, can effectively restore the physiological curvature of the lumbar spine and provide good stability. However, for patients with osteoporosis, the holding force of conventional pedicle screws is greatly reduced owing to the reduction of vertebral bone mass and structural degeneration, and internal fixation cannot provide effective stability. Bone cement-enhanced pedicle screw fixation is a commonly used internal fixation enhancement technique in patients with osteoporosis. After the pedicle screw is implanted, it passes through the hollow channel inside the pedicle screw and the hole at the distal end of the screw. A certain amount of diluted bone cement was injected to diffuse the bone cement at the distal end of the screw to increase the contact area between the screw and the vertebral body and increase the stability of the screw fixation. Studies have shown that, in osteoporotic vertebral bodies, the anti-extraction force of bone cement-enhanced pedicle screws is 2 to 5 times that of traditional screws.^[1]^ Although this technology has been widely used in clinical practice, there are an increasing number of reports on its related complications. Similar to percutaneous vertebroplasty (PVP) and percutaneous kyphoplasty (PKP), the most common complication associated with this internal fixation method is bone cement leakage. Although most patients are asymptomatic, leakage of bone cement into the spinal canal, intradural, nerve root outlets, etc, can cause severe spinal cord nerve damage, and even lead to pulmonary embolism and intracardiac embolism through the peripheral venous system, and produce serious complications, endangering the patient's life and safety.^[2–4]^ In this report, we report the case of a patient with osteoporosis and degenerative disease of the lumbar spine who had intracardiac cement embolism after posterior decompression and cement-enhanced pedicle screw fixation, resulting in severe right atrium and right ventricle foreign bodies, anterior tricuspid valve damage, and some chordae rupture.

## 2 Case report

**Figure 1:**
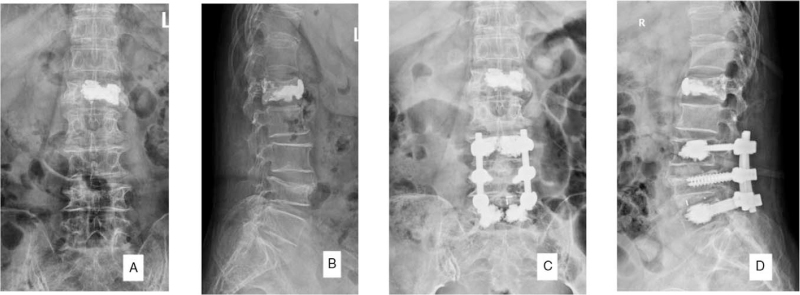
Comparison before and after treatment of osteoporosis and degenerative lumbar spine with posterior lumbar decompression and bone cement enhanced pedicle screw fixation.

**Figure 2:**
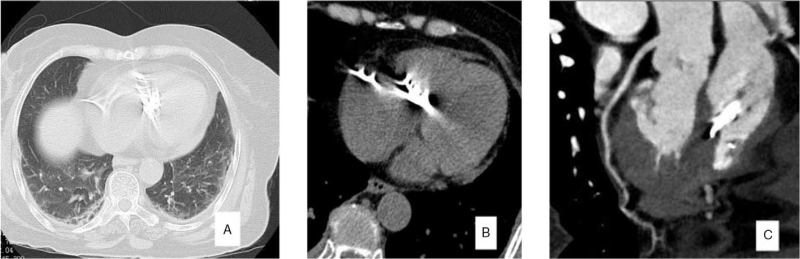
CT and coronary angiography of the chest showed multiple compact and strip-shaped images in the heart.

A 70-year-old female patient with previous osteoporosis and L1 vertebral compression fracture underwent PVP surgery. This time, she was admitted to the hospital because of thickening of the L3/4 and L4/5 bilateral ligamentum flavum and spinal stenosis. Eliminating the surgical contraindications, posterior lumbar decompression and cement-enhanced pedicle screw fixation were performed (Fig. 1A--D). On the night after surgery, the patient developed chest tightness, blood oxygen saturation decreased, the measured value of high-flow oxygen inhalation was 92%, and there were no obvious abnormalities in cardiopulmonary auscultation. The patient's myocardial infarction serial examination showed: creatine phosphate kinase: 511 U/L, lactate dehydrogenase: 341 U/L, high-sensitivity Troponin I: 0.809 ng/mL; after 4 hours, the high sensitivity troponin I rose to 0.945 ng/mL; The electrocardiogram showed sinus tachycardia, ST-T change, Compared with the ECG of 2021-7-13, the ST segment moved down compared to the front, and the T wave changed from upright to low-flat/inverted. Symptoms did not improve after symptomatic treatment. Therefore, we suspected that pulmonary embolism or myocardial infarction was possible. Cardiac color Doppler ultrasound examination: Heart color Doppler ultrasound showed strong echo in the right ventricle and partial embedding in the myocardium. The right atrium and ventricle were enlarged with severe tricuspid regurgitation. Chest computed tomography (CT) examination showed (Fig. 2A,B) multiple sheets and strips of dense shadows in the heart. Coronary artery CT revealed the following (Fig. 2C): coronary arteriosclerosis, slight stenosis of the proximal LAD lumen; there was little pericardial effusion observed; and high-density shadows in the heart valve area.

**Figure 3:**
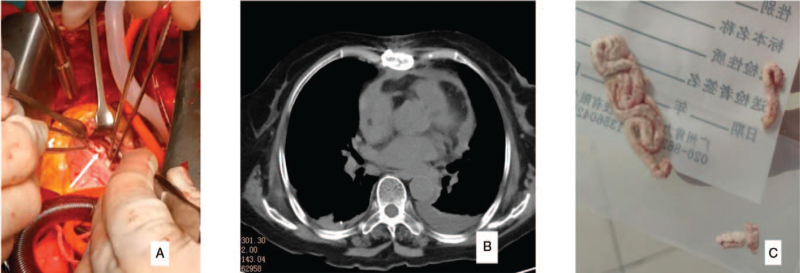
Intraoperative photos (arrows indicate bone cement) and postoperative CT scan of the chest, showing that the bone cement has been taken out; C is the bone cement taken out.

After an emergency consultation with a multidisciplinary team composed of cardiac and vascular surgeons, we concluded that intracardiac cement embolism occurred through the vertebral vein after bone cement leakage. After multidisciplinary team discussion composed of cardiac surgeons and vascular surgeons, the patient decided to undergo the removal of intracardiac foreign bodies under cardiopulmonary bypass and tricuspid valvuloplasty (Fig. 3A). The right atrium was incised, and inspection revealed that the cord-shaped foreign body was embedded in the wall of the right atrium, 15 mm_∗_3 mm in size with hard texture. A long irregular foreign body was seen in the right ventricle, 40 × 10 mm in size, hard, and embedded in the tricuspid valve. The anterior tricuspid valve was damaged and the chordae was ruptured. The foreign bodies were gently removed (Fig. 3C), and the right atrium, right ventricle, and entrance of the pulmonary artery were explored. No foreign body was observed. Tricuspid valvuloplasty was performed with repair of the chordae. Edward 28 “C” valve annulus with double-ended polyester thread interrupted mattress suture was performed for tricuspid valvuloplasty. On injecting water, it was observed that the tricuspid repair was not satisfactory. The valve annulus was then removed and the 4/0 prolene line (Devega method) was used to contract the tricuspid valve. Water injection showed that the tricuspid valve closure was better than before. The incision of the right atrium was sutured with a 5/0 Prolene continuous suture. Chest radiography was performed using the C-arm machine tool, and no foreign body was found in the heart. Intraoperative transesophageal color Doppler ultrasound showed no foreign bodies in the heart. Incomplete severe lateral regurgitation of the anterior tricuspid valve was noted. The right atrium was then reopened, the outside of the anterior tricuspid valve was repaired under the beating heart, and the right atrium incision was sutured. The reassessment of cardiac color Doppler ultrasound indicated that tricuspid regurgitation was significantly reduced. Cardiopulmonary bypass was stopped, the tubes were removed, protamine-neutralized heparin was administered, and bleeding was carefully stopped. Symptomatic and supportive treatment was given after the operation. The patient recovered and was discharged after 3 weeks. There was no intracardiac foreign body on chest CT after the operation (Fig. 3B) and high-sensitivity troponin I before discharge (0.061 ng/mL).

## 3 Discussion

Currently, bone cement-enhanced pedicle screw fixation is the current standard treatment method for osteoporotic lumbar degenerative diseases. The anti-extraction force of the pedicle screw is enhanced by injecting bone cement into the built-in channel of the pedicle screw. The stability of internal fixation can effectively improve patients’ quality of life. However, like PVP and PKP, cement-enhanced pedicle screw fixation also faces complications including bone cement extravasation, pulmonary embolism, infection, and spinal cord nerve injury. Studies have found that 72% to 82% of bone cement leakage has no significant clinical symptoms, but part of the leakage can enter the lungs and heart through the peripheral venous system.^[5,6]^ For patients with more leakages, this may cause serious health damage and even fatal consequences. Clinically reported risk factors for cement leakage include high injection pressure, low cement viscosity, pathological fractures (osteoporotic or neoplastic), high-dose injection of bone cement, and defects in the posterior wall of the vertebral body.^[7–11]^ With the advancement of clinical operation techniques, surgical operations to prevent bone-cement leakage have also been highly developed.

According to the analysis of most literature, the discovery of bone cement leakage is mainly due to accidental findings in routine inspections. Most reports mainly rely on routine X-ray examinations after surgery instead of CT scans, which, to some extent, leads to the missed diagnosis of bone cement leakage. In clinical diagnosis and treatment, asymptomatic bone cement leakage is regarded as a normal phenomenon of surgical treatment because it does not require further treatment and is, therefore, ignored by clinicians. Some scholars have suggested that CT scans should be performed after surgery to accurately assess bone cement leakage.^[12,13]^ Janssen et al^[14]^ found that 64.8% of patients who underwent cement-enhanced pedicle screw fixation had intravascular cement leakage. The authors believe that compared with PVP, bone cement-enhanced pedicle screw fixation has a higher risk of intravascular cement leakage. This is because compared with PVP, cement-enhanced pedicle screws require more bone cement, and the total amount of injection is also higher. For pulmonary embolism and intracardiac embolism caused by bone cement leakage, the potential harm is huge and needs to be actively dealt with in clinical work. For patients with intracardiac embolism, it is necessary to complete chest CT and coronary CT examinations to assess damage to the heart, valves, chordae, and coronary artery. The distribution of bone cement should be determined to provide a reference for further surgical treatment. Conventionally, for patients with valve damage and coronary stenosis, open-heart surgery is the first choice,^[15,16]^ which fully removes the embolized bone cement and repairs the damaged heart valves and blood vessels.

In our case, although the patient did not have vertebral body fracture, intraoperative nail placement did not damage the vertebral body wall. After the operation, the patient did not have obvious symptoms of chest tightness or chest pain but only showed low blood oxygen saturation. Electrocardiography showed abnormal myocardial enzymes, and chest CT and coronary angiography were abnormal. It is necessary to be highly vigilant against the possibility of pulmonary embolism and intracardiac embolism because bone cement can enter the peripheral venous system through the paravertebral venous system and migrate to the lungs and heart. Early chest CT is an important method for determining whether bone cement pulmonary embolism and intracardiac embolism occur. In cases of intravascular foreign body embolism, well-organized multidisciplinary teamwork is very important.^[17]^ We organized a multidisciplinary team composed of cardiac and vascular surgeons to conduct a combined operation after thorough discussions and achieved ideal treatment results.

## 4 Conclusion

Similar to PVP and PKP, bone cement enhanced pedicle screw fixation can produce the same bone-cement leakage complications, and most patients are asymptomatic. It is worth noting that for patients with postoperative cardiopulmonary discomfort, in view of the limitations of radiographic examination, we recommend performing chest CT examination to confirm the diagnosis. Patients with embolism of foreign bodies in blood vessels require joint treatment by a multidisciplinary team to save their lives.

## Acknowledgments

We thank the patient and his family for agreeing to report this case report. Thanks to the vascular interventional department and cardiothoracic surgery experts of our hospital, they provided the patient with a good cardiac operation and saved the patient's life.

## Author contributions

**Conceptualization:** Kui Sun, Biru Liang.

**Data curation:** Kui Sun, Fuli Huang, Biru Liang.

**Formal analysis:** Kui Sun, Biru Liang.

**Investigation:** Kui Sun, Fuli Huang, Biru Liang.

**Methodology:** Biru Liang.

**Project administration:** Kui Sun, Biru Liang.

**Resources:** Kui Sun.

**Supervision:** Fuli Huang, Biru Liang.

**Visualization:** Kui Sun.

**Writing – original draft:** Kui Sun.

**Writing – review & editing:** Kui Sun, Fuli Huang, Biru Liang.
